# Supplementary material for: A validated lineage-derived somatic truth data set enables benchmarking in cancer genome analysis
Source: Commun Biol. 2020 Dec 8;3:744. doi: 10.1038/s42003-020-01460-9 (PMC7722876; doi:10.1038/s42003-020-01460-9)
Supplement: Supplementary file 2 — Reporting Summary [file 42003_2020_1460_MOESM2_ESM.pdf]

nature research

Corresponding author(s): Megan Shand

Last updated by author(s): 10/18/2020

Reporting Summary

Nature Research wishes to improve the reproducibility of the work that we publish. This form provides structure for consistency and transparency in reporting. For further information on Nature Research policies, see Authors & Referees and the Editorial Policy Checklist .

Statistics

For all statistical analyses, confirm that the following items are present in the figure legend, table legend, main text, or Methods section.

n/a	Confirmed	
☐	☒	The exact sample size (*n*) for each experimental group/condition, given as a discrete number and unit of measurement
☐	☒	A statement on whether measurements were taken from distinct samples or whether the same sample was measured repeatedly
☐	☒	The statistical test(s) used AND whether they are one- or two-sided<br>*Only common tests should be described solely by name; describe more complex techniques in the Methods section.*
☒	☐	A description of all covariates tested
☒	☐	A description of any assumptions or corrections, such as tests of normality and adjustment for multiple comparisons
☐	☒	A full description of the statistical parameters including central tendency (e.g. means) or other basic estimates (e.g. regression coefficient) AND variation (e.g. standard deviation) or associated estimates of uncertainty (e.g. confidence intervals)
☐	☒	For null hypothesis testing, the test statistic (e.g. *F*, *t*, *r*) with confidence intervals, effect sizes, degrees of freedom and *P* value noted<br>*Give P values as exact values whenever suitable.*
☒	☐	For Bayesian analysis, information on the choice of priors and Markov chain Monte Carlo settings
☒	☐	For hierarchical and complex designs, identification of the appropriate level for tests and full reporting of outcomes
☐	☒	Estimates of effect sizes (e.g. Cohen's *d*, Pearson's *r*), indicating how they were calculated

Our web collection on statistics for biologists contains articles on many of the points above.

Software and code

Policy information about availability of computer code

Data collection	No software was used for data collection.
Data analysis	BWA-MEM-0.7.15, Picard-2.18.15, GATK-4.0.10.1 (primary analysis), GATK-4.1.2.0 (CNV analysis and Mutect2), GATK-4.1.1.0 (mitochondria analysis), GATK-4.0.4.0 (HaplotypeCaller and VQSR), GATK-4.1.7.0 (Funcotator), Custom GATK fork (https://github.com/meganshand/gatk), GATK-3.8-1-0 (CombineVariants), Samtools-1.3.1, Strelka-2.9.2, VarScan-2.3.9, and RTG Tools-3.9.1 were used for data analysis.

For manuscripts utilizing custom algorithms or software that are central to the research but not yet described in published literature, software must be made available to editors/reviewers. We strongly encourage code deposition in a community repository (e.g. GitHub). See the Nature Research guidelines for submitting code & software for further information.

Data

Policy information about availability of data

All manuscripts must include a data availability statement. This statement should provide the following information, where applicable:
- Accession codes, unique identifiers, or web links for publicly available datasets
- A list of figures that have associated raw data
- A description of any restrictions on data availability

Illumina reads aligned to hg19[GRCh37] are available in SRA under accession number SRP159787. All other data (Illumina reads aligned to GRCh38, variant calls, and confident regions) can be acquired from https://app.terra.bio/#workspaces/broad-dsp-spec-ops-fc/somatic_truth_data_from_cell_lineage with a free account.

Field-specific reporting

Please select the one below that is the best fit for your research. If you are not sure, read the appropriate sections before making your selection.

- [x] Life sciences
- [ ] Behavioural & social sciences
- [ ] Ecological, evolutionary & environmental sciences

For a reference copy of the document with all sections, see nature.com/documents/nr-reporting-summary-flat.pdf

Life sciences study design

All studies must disclose on these points even when the disclosure is negative.

Sample size	One LinSeq experiment was chosen which included 11 leaf samples.
Data exclusions	No data were excluded from the analysis.
Replication	All possible pairings of samples were used to generate benchmarking data to ensure that the variance between sequenced samples did not affect our general conclusion.
Randomization	No randomization was applied because we did not partition the data.
Blinding	Not applicable because we did not partition the data.

Reporting for specific materials, systems and methods

We require information from authors about some types of materials, experimental systems and methods used in many studies. Here, indicate whether each material, system or method listed is relevant to your study. If you are not sure if a list item applies to your research, read the appropriate section before selecting a response.

Materials & experimental systems

n/a	Involved in the study
☒	☐ Antibodies
☐	☒ Eukaryotic cell lines
☒	☐ Palaeontology
☒	☐ Animals and other organisms
☒	☐ Human research participants
☒	☐ Clinical data

Methods

n/a	Involved in the study
☒	☐ ChIP-seq
☒	☐ Flow cytometry
☒	☐ MRI-based neuroimaging

Eukaryotic cell lines

Policy information about cell lines

Cell line source(s)	HT115 was purchased from the Broad Cancer Cell Line Encyclopedia.
Authentication	Cell identity was authenticated by the Broad Genomics Platform for HT115 using previously stored fingerprint genotypes. The fingerprint consists of the genotype at 82 loci from the query sample which were compared against fingerprints of all the Cancer Cell Line Encyclopedia cell lines using the farthest neighbor graph (FNG) algorithm. The highest correlation (0.83) was found between our HT115 sample and the CCLE HT115 sample.
Mycoplasma contamination	No Mycoplasma contamination observed in sequence data.
Commonly misidentified lines (See ICLAC register)	No commonly misidentified cell lines were used.
